# An assessment of the accuracy and availability of data in electronic patient tracking systems for patients receiving HIV treatment in central Mozambique

**DOI:** 10.1186/1472-6963-12-30

**Published:** 2012-02-02

**Authors:** Barrot H Lambdin, Mark A Micek, Thomas D Koepsell, James P Hughes, Kenneth Sherr, James Pfeiffer, Marina Karagianis, Joseph Lara, Stephen S Gloyd, Andy Stergachis

**Affiliations:** 1 Pangaea Global AIDS Foundation, Oakland, CA, USA; 2 Department of Global Health, University of Washington, Seattle, WA, USA; 3 Health Alliance International, Seattle, WA, USA; 4 Department of Epidemiology, University of Washington, Seattle, WA, USA; 5 Department of Health Services, University of Washington, Seattle, WA, USA; 6 Department of Biostatistics, University of Washington, Seattle, WA, USA; 7 Ministry of Health, Mozambique, Beira, Sofala, MZ; 8 Health Alliance International, Beira, Sofala, MZ; 9 Global Medicines Program, University of Washington, Seattle, WA, USA

## Abstract

**Background:**

Since the rapid scale-up of antiretroviral therapy (ART) programs in sub-Saharan Africa, electronic patient tracking systems (EPTS) have been deployed to respond to the growing demand for program monitoring, evaluation and reporting to governments and donors. These routinely collected data are often used in epidemiologic and operations research studies intended to improve programs. To ensure accurate reporting and good quality for research, the reliability and completeness of data systems need to be assessed and reported. We assessed the completeness and reliability of EPTS used in 16 HIV care and treatment clinics in Manica and Sofala provinces of Mozambique.

**Methods:**

We conducted a cross-sectional study to assess the completeness and reliability of key variables in the electronic data system for patients enrolling in 16 public sector HIV treatment clinics between 1 July 2004 and 30 June 2008. Data from the electronic database was compared with data abstracted from a stratified random sample of 520 patient charts. Percent agreement, kappa scores and concordance correlation coefficients were calculated for specified variables. Percentile bootstrap confidence intervals were calculated to account for the stratified nature of our sampling.

**Results:**

A total of 16,149 patients with a median age of 33 years and a median CD4 count of 151 enrolled in these 16 clinics between 1 July 2004 and 30 June 2008. The level of completeness was high for most variables with height (18.6%) and weight (11.5%) having the highest amount of missing data. The level of agreement for available data was also high with reliability statistics of 0.95 (95% CI: 0.92-0.98) for gender, 0.91 (95% CI: 0.80-1.00) for pre-ART CD4 value and 0.97 (95% CI: 0.95-0.99) for patient retention.

**Conclusions:**

Electronic patient tracking systems have been deployed to respond to the growing monitoring, evaluation and reporting requirements. In our cross-sectional study of clinics in Manica and Sofala provinces of Mozambique, we found high levels of completeness and reliability for key variables indicating that these electronic databases provided adequate data not only for monitoring and evaluation but also for research. Routine evaluations of the completeness and reliability of these databases need to occur to ensure high quality data are being used for reporting and research.

## Background

As antiretroviral treatment (ART) programs have been rapidly scaled up in sub-Saharan Africa, electronic data systems have been introduced to support program monitoring and evaluation imperatives [1]. These data systems have facilitated the response to the growing demands of patient management and reporting to governments and donor groups specific for HIV care and treatment delivery, and they rely on complete and accurate charting by providers of laboratory, pharmacy and medical visits into patient charts. They were developed in parallel to national data systems due to the inadequacy of existing in-country information systems and data collection mechanisms [1]. Typically in resource-limited settings, program metrics for reporting are hand calculated on a monthly basis from paper-based registries for a given health facility which are then aggregated at the district, provincial and national-level [1,2]. Donor agencies require information on not only the number of persons accessing services but also estimates of patient follow-up and retention at different points in time as well as other performance indicators [3]. HIV care and treatment is a chronic intervention that requires, in addition to the typical program reporting requirements, proper treatment and monitoring of patients over many years. Effective delivery of HIV care and treatment over time requires timely information on the quantity and quality of care being provided by the workforce to patients. These data are vital to adequately monitor site performance and inform national planning [4].

Researchers have used electronic patient tracking systems in epidemiologic and operations research studies of ART programs [5-10]. However, only one of these studies provides results regarding the reliability of data [10]. In order to use these data systems for epidemiologic and operations research and to ensure accurate reporting of statistics to government agencies and donors, the reliability and completeness of data systems in different settings needs to be assessed and reported.

In Manica and Sofala provinces of Mozambique, an electronic patient tracking system (EPTS) assisted 18 HIV care and treatment clinics in monitoring patients' clinical, pharmaceutical, and follow-up procedures and in reporting aggregated performance data to government and donor agencies. The EPTS was first implemented in these sites, due to the length of time the clinics had been open and the volume of patients seen, as part of a regional roll-out of the data system. The EPTS also provides a potential source of data to assess both patient and clinic-level characteristics within the Mozambique public health system. We conducted a cross-sectional study to assess the accuracy of key variables in the EPTS compared to the source data recorded in patients' charts and evaluated the availability of data in the EPTS relevant to the study of patient attrition.

## Methods

### Study Setting

In 2007, the population totalled 1.65 million in Sofala and 1.42 million in Manica with nearly 22% of the population based in the capitals of Beira and Chimoio [11]. At the end of 2009 an estimated 11.5% of Mozambique's population was infected with HIV. Central Mozambique had one of the heaviest HIV burdens in the country: 15.5% prevalence in Sofala; 15.3% in Manica [12]. In 2003, the first ART delivery site in the two provinces opened in Sofala's central hospital while a second one opened in 2004. To improve access to HIV care and treatment, the Ministry of Health integrated ART services into primary health care clinics distributed throughout Manica and Sofala provinces.

HIV-infected patients who enrolled into HIV care were assessed for ART eligibility. The initial visit included a documentation of demographic characteristics, a medical history/physical exam where patients were clinically staged according to WHO criteria and a blood sample was drawn for a CD4 cell count. Patients were then determined to be eligible for ART if they had a CD4 cell count below 200 cells/μL regardless of WHO Stage, a CD4 count below 350 cells/μL if in WHO Stage III or pregnant, or were in WHO Stage IV regardless of CD4 count [13].

Once a patient was eligible for ART, the patient was referred to a social worker for several sessions of pre-ART counseling, and ART was subsequently initiated. Three current first line regimens were used, each using fixed-dose combination pills. Triomune (D4T+3TC+NVP) was the first regimen of choice. Patients who were concurrently being treated for Tuberculosis (TB) were given Lamivir-S (D4T+3TC) and Efavirenz (EFV). Patients who were pregnant were given Combivir (AZT+3TC) and NVP [13].

After ART initiation, patients returned on a periodic basis for clinical and laboratory monitoring and on a monthly basis for pharmacy refills, according to national standards [13]. Clinic-based social workers also provided adherence support as needed. After a patient failed to return for ART medication refill, *activistas *(clinic-based peer counselors) were notified and began actively tracing patients. The peer counselors visited the patient's residence and encouraged them to return for treatment at the clinic if the patient was still alive. If the patient had died, the date of death was documented. These procedures were standardized across all clinics included in the study.

Mozambique's national health information system (HIS) operates much like other national data systems in the region and incorporates both paper and electronic components. As mentioned above, paper-based registries are utilized in health facilities to track the number of people coming in for services on a daily basis. Specific indicators are then tabulated on a monthly basis and aggregated into monthly facility reports. These reports are transferred to the district-level where the district planning and statistics department typically enters the monthly numbers by facility into the Ministry of Health's HIS, referred to as the *Modulo Basico*. Electronic files are then sent to the provincial level where they are pulled together and forwarded to the national level. Attempts to evaluate the completeness, reliability and validity of the HIS in this region are ongoing [14]. Mozambique's HIS has not been modified to respond to the growing need to have patient-level data systems for HIV care and treatment.

### Electronic Patient Tracking System

In Manica and Sofala provinces, HIV clinics utilized a Microsoft Access-based EPTS containing demographic, clinical and pharmaceutical data of patients enrolled into the HIV care and treatment system (Table 1). The implementation of EPTS into different clinics was a programmatic decision which factored in how long clinics had been open (those open longer tended to be prioritized) and whether they had a dedicated data technician. The database included an administrative file table containing demographic variables and socioeconomic factors and a health services file table that included pharmaceutical and clinical data. All patient-level data were data elements in the national HIV patient medical records and were also linkable through a unique patient identification number.

**Table 1:** Variables collected in the Electronic Patient Tracking System

Administrative	Health Services
-Clinic identification number	-Patient identification number
-Patient identification number	-Date of visit
-Date of enrollment at HIV clinic	-Type of provider seen
-Pregnancy status at time of enrollment	-Pregnant at time of visit
-Number of months pregnant at enrollment	-Temperature
-Age	-Weight
-Gender	-Height
-Site of HIV testing	-CD4 count
-Date of HIV testing	-CD4 percentage
-Results of HIV testing	-Date of CD4 count
-City of residence	-Viral load
-Neighborhood of residence	-Date of viral load
-Marital status	-Clinical diagnoses*
-Type of employment	-WHO stage
-Years of education	-CTX prophylaxis
-Type of higher education	-TB prophylaxis
-Patient active at HIV clinic (yes/no)	-Patient receiving ART
-Reason patient left HIV clinic	-Type of ART regimen
-Date patient left HIV clinic	-Type of ART refill visit
-Date the HIV clinic found out patient left	-# of pills dispensed
-Exit comments	-Next refill date
-ART committee date	-Receiving TB treatment
-Patient receiving ART (yes/no)	-Comments
-ART start date	
-Type of ART initiation (new patient or transferred from another facility)	
-Comments about treatment	
-Pregnancy status at time of treatment initiation	
-Prophylaxis for PMTCT	
-Prophylaxis type	
-Prophylaxis date	

*includes 63 clinical diagnoses; HIV - Human Immunodeficiency Virus; ART - Antiretroviral Therapy; WHO - World Health Organization; PMTCT - Prevention of Mother to Child Transmission; CTX - Cotrimaxazole; TB - Tuberculosis; CD4 - Cluster of Differentiation 4

During the initial visit for HIV-infected patients enrolling into HIV care, demographic and clinical characteristics were recorded in the patient charts. In addition, a CD4 count blood specimen was sent to the laboratory, and after processing, the results were recorded in the patient's chart. When a patient initiated HIV treatment and for each subsequent pharmacy visit, the ART regimen, date of dispensing and next refill date were recorded. These data were first recorded in the patient's chart by providers and then were entered by the data technician into the EPTS via standardized data entry forms after the initial visit and after each of the subsequent clinic and pharmacy visits.

The clinics had standardized treatment and patient tracking protocols as all of them were managed by Mozambique's Ministry of Health with technical and logistical support provided by Health Alliance International. Databases were maintained by data technicians, while managers provided on-the-job training and quality control. Data technicians were located at the health facilities and were responsible for transcribing patient charts into the EPTS on a real-time basis. The EPTS had numerous range checks and cross-checks to enable immediate detection and correction of data entry errors. Database managers worked at the provincial-level and monitored data quality by running queries and troubleshooting with technicians if discrepancies existed. The managers circulated throughout the province to work with each of the technicians located at health facilities. In addition, the managers were responsible for producing quarterly, semi-annual and annual reports of program statistics.

### Study Design

To evaluate the reliability of the EPTS, we conducted a cross-sectional study among adult (≥ 15 years of age), ART-naïve patients receiving HIV treatment in 16 HIV care and treatment centers between July 1, 2004 and June 30, 2008 in Manica and Sofala provinces of Mozambique. Data from the EPTS had been previously compared to patient chart data for two of the 18 clinics and thus these two clinics were not included in this assessment [10]. We compared patient-level information contained in the EPTS with data abstracted from a total of 520 patient charts. Stratified, random sampling was utilized to select patient charts. Strata were defined by the clinic where patients currently received HIV treatment, and the number of patient charts targeted for a particular clinic depended on the size of the patient population receiving HIV care: 20 charts for clinics with < 200 patients, 30 charts for clinics with 200 to 499 patients and 40 charts for clinics with ≥ 500 patients. A total of 520 patient charts were surveyed. Assuming α = 0.05, a standard error of 0.0136 (based on observed data incorporating stratified sampling), our margin of error was ± 2.67% around an expected kappa score of 90%. Using a random list of patient identification numbers, trained data collectors abstracted demographic, clinical and pharmaceutical data from structured patient charts including gender, pre-ART CD4 count, date of CD4 count, first pharmacy pick-up date of antiretroviral medicines at treatment initiation, retention of the patient in HIV care and treatment (patient retention) and outcome date as described below. Pilot testing indicated that abstractors tended to make errors in identifying the correct pieces of information while blinded to the EPTS values due to the complexity of patient charts. Therefore, data abstractors were not blinded to the EPTS values. Patient retention was defined as patients who either remained in the program, suspended treatment per clinician's recommendation but remained in care or transferred to receive treatment at another facility. Patients who had died or were lost to follow-up for reasons other than death were considered to be not retained in the program. The patient's outcome date was classified as the last pharmaceutical refill date for patients who remained in the program or who were lost to follow-up for reasons other than death, and the date of death, suspension or transfer was used as the outcome date for those who had died, suspended or transferred.

In addition, we evaluated the availability of baseline data for all adult, ART-naïve patients who initiated HIV treatment for data commonly used in the study of attrition from HIV treatment programs: age, gender, education, weight, height, WHO stage and CD4 count [7,8,10,15-17].

### Statistical Methods

Data from the standardized patient charts were compared to data contained in the EPTS. Several measures were used to assess the extent to which the EPTS accurately reflected the data in patients' charts for the sample of 520 patients. For binary variables, percent agreement and kappa scores were generated [18]. Concordance correlation coefficients were used to estimate the degree of agreement for continuous variables [19]. For dates, we calculated the percent agreeing exactly and the percent agreeing within 7 days of the date recorded in the patient chart. We calculated percentile bootstrap confidence intervals based on 1,000 replications for percent agreement, kappa scores and concordance correlation coefficients. The bootstrapping accounted for the stratified nature of our sampling for kappa scores and concordance correlation coefficients. To be consistent, we also used the approach for percent agreement. All analyses were conducted in Stata version 11.1 (College Station, TX).

The study was approved by the ethical review committees of the Mozambique Ministry of Health (Comité Nacional de Bioética Para Saúde; Reference: 337/CNBS) and the University of Washington Human Subjects Division (Reference: 30410).

## Results

### Clinic Characteristics

A total of 16 of the 36 HIV care and treatment clinics recognized by the Ministry of Health from Manica and Sofala provinces were included in the analysis. Two of the 36 clinics were included in a previous assessment, and the remaining 18 were not included since they did not have electronic patient tracking systems. Two of these clinics were a vertical, stand-alone HIV care and treatment clinics, 14 were clinics where HIV care and treatment was integrated with primary health care services; and one of the vertical model clinics shifted into an integrated model. In addition, 7 (44%) of the clinics were located in urban settings. Four (25%) were considered to be hospitals while 12 (75%) were considered to be health centers.

### Availability of Baseline Characteristics in the EPTS

The baseline characteristics of patients enrolling into HIV treatment between 1 July 2004 and 30 June 2008 are displayed in Table 2. Overall, 16,149 adult (≥15 years of age) patients enrolled for antiretroviral therapy in these 16 clinics with a median age of 33 years and a median CD4 count of 151. For these patients, the level of completeness was high for most of the variables with WHO Stage (9.9%), weight (11.5%) and height (18.6%) having the highest amounts of missing data. Of the 520 charts reviewed, 19 (3.7%) had missing CD4 counts and dates in the database, 7 (1.3%) also had missing CD4 information in the patient charts.

**Table 2:** Availability of Baseline Characteristics among Patients Initiating HIV Treatment in the Electronic Patient Tracking System

	**Patients in Treatment** **N = 16,149**	**Missing** **N (%)**
	
**Age (years), Median (IQR)**	33 (27-40)	38 (0.2)
**Education (years), Median (IQR)**	6 (3-8)	1,194 (7.4)
**Gender, N (%)**		
Male	6,345 (39.3)	1 (0.01)
Female	9,803 (60.7)	
**CD4 Count, Median (IQR)**	151 (84-226)	726 (4.5)
**WHO Stage, N (%)**		
Stage I	1,927 (11.9)	1,597 (9.9)
Stage II	2,353 (14.6)	
Stage III	8,903 (55.1)	
Stage IV	1,369 (8.5)	
**Weight (kg), Median (IQR)**	50.5 (45.0-57.0)	1,857 (11.5)
**Height (m), Median (IQR)**	1.61 (1.56-1.68)	3,000 (18.6)

### Reliability of Data in the EPTS

A total of 520 patient charts were reviewed to evaluate the reliability of specific variables in the electronic patient tracking system. All of the variables had a high level of agreement and reliability. Patient retention in the EPTS had the highest level of agreement [99.23% (95% CI: 98.46-99.81)] with a kappa score of 0.97 (0.95-0.99). The date of CD4 count at treatment initiation had the lowest level of agreement [92.61% (95% CI: 90.42-94.61)] while 97.6% (95% CI: 96.41-98.80) agreed within 7 days of the CD4 date located in the patient's chart. Among those records with discordant CD4 dates, the average difference was 27.05 days with a median of 1 day (IQR: 1-10). Of clinical significance, the value of the CD4 count at baseline also had high levels of agreement and reliability (Table 3).

**Table 3:** Reliability of Data for Patients Entering HIV Treatment in EPTS

	**% Agree** **(95% CI)**	Reliability Statistic* (95% CI)
	
**Gender**	97.69 (96.35-98.85)	0.95 (0.92-0.98)
**CD4 Value**	98.40 (97.21-99.40)	0.91 (0.80-1.00)
**Patient Retention†**	99.23 (98.46-99.81)	0.97 (0.95-0.99)
**CD4 Date**	92.61 (90.42-94.61)	-
**ART Initiation Date**	97.88 (96.72-99.04)	-
**Outcome Date**	95.91 (94.35-97.47)	-

*Kappa score for gender and patient retention; Concordance correlation coefficient for CD4 value; No reliability statistic for date variables since they are highly dependent on the scale of the continuous variable which is arbitrary for date values. †As of July 2008

Regarding the ART initiation date of patients, 97.88% (95% CI: 96.72-99.04) of the records agreed to the exact day while 98.84% (95% CI: 97.88-99.61) agreed within 7 days of the date recorded in patient charts. The mean difference for those with discrepant ART initiation dates was 26 days with a median difference of 13 (IQR: 2-26). The outcome date agreed 95.91% (95% CI: 94.35-97.47) of the time while 98.05% (95% CI: 96.88-99.02) agreed within 7 days of the date recorded in the patient's chart. For those patients with discordant outcome dates, the mean difference was 14 days with a median of 7 days (IQR: 2-17).

## Discussion

Electronic patient tracking systems have evolved to respond to the growing demands of patient management and program reporting specific to HIV care and treatment delivery. In this cross-sectional study to determine the reliability of data within the EPTS for 16 HIV care and treatment clinics in central Mozambique, we found a high level of agreement between patient charts and the EPTS. Moreover, the EPTS contained relatively complete baseline demographic and clinical information for patients in antiretroviral therapy.

In another cross-sectional study of the same EPTS conducted in two large urban hospitals in Beira and Chimoio, Mozambique, 300 patient charts, also selected as a stratified random sample, were reviewed to compare data to that in the EPTS. Briefly, in that study the percent agreement was 99.5% (Kappa = 0.98) for patient gender, 91.8% (Kappa = 0.83) for provider type seen at first visit, 97.5% for CD4 date pre-treatment initiation, 95.2% (Kappa = 0.91) for CD4 count pre-treatment initiation, 97.5% (Kappa = 0.94) for whether a CD4 count was done 6 months post-treatment initiation and 95.7% (Kappa = 0.90) for the CD4 count 6 months post-treatment initiation^8^. These results demonstrated 'almost perfect' levels of agreement in the EPTS for these 2 vertical model HIV care and treatment clinics which have the largest patient populations in Manica and Sofala provinces. Our study also found 'almost perfect' levels of agreement among a greater number of clinics which included those clinics where HIV care and treatment was integrated into primary health care clinics spread throughout the two provinces [15]. In addition, our study showed very high levels of completeness with most variables; of the 19 (4%) CD4 counts and dates that were missing in the EPTS, 63% of them were a result of improper transcription from the patient charts to the database by the data technician. However, given that some variables had missing data, program managers may use this information to improve recording further through data feedback at the clinic level.

One of the limitations of our study is that we did not randomly select variables to evaluate. We selected indicators that have been emphasized by program managers as key variables to providers and data technicians and are frequently used in program reporting as well as epidemiologic and operations research studies. Appropriate recording of patient data in charts with standardized forms from the Ministry of Health had been highlighted with providers as a necessary part of high quality data. A number of important variables such as cotrimoxazole prophylaxis as well as diagnosis and treatment of other co-morbidities were not included in this study as previous assessments had shown that availability of these data in the EPTS was low due, in part, to poor charting and the unavailability of standardized forms. In addition, our study did not have sufficient power to look at differences between clinics or clinic characteristics.

Strengths of the study include the standardization of data recording and data assessment protocols across the clinics included in the study. Additionally, we were able to assess the reliability and availability of data with a large number of clinics and this study was the largest of its kind to date in this setting.

Our study showed that the EPTS for these 16 clinics contained data that were relatively complete with a high level of agreement with patients' medical records. These databases provide adequate data to properly monitor patients, report to donors and governmental agencies and conduct epidemiologic and operations research. Future studies should focus on monitoring the availability and accuracy of data regarding the clinical monitoring process for patients (i.e. CD4 count at 6 and 12 months post treatment initiation) and be designed to capture the potential variability between clinics.

## Conclusions

The electronic patient tracking systems for HIV treatment programs in Manica and Sofala provinces of Mozambique had high levels of completeness and reliability justifying their use not only for program reporting to the Ministry of Health and donors but also for epidemiologic and operations research. As electronic data systems are used more widely to support HIV treatment programs, researchers should formally evaluate and report levels of completeness and reliability of these systems to ensure that high quality data are being used for both reporting and research. Future initiatives should focus on both improving these systems to fill in the data gaps and continuously monitoring the data quality.

## List of Abbreviations

ART: Antiretroviral Therapy; HIV: Human Immunodeficiency Virus; WHO: World Health Organization; EPTS: Electronic Patient Tracking System; CD4: Cluster of Differentiation 4; CI: Confidence Interval; IQR: Interquartile Range; KG: Kilograms; M: Meters; TB: Tuberculosis; CTX: Cotrimaxazole; PMTCT: Prevention of Mother to Child Transmission

## Competing interests

At the time of the study, the following authors were employed by Health Alliance International: Barrot Lambdin, Mark Micek, Kenneth Sherr, Joe Lara, James Pfeiffer and Stephen Gloyd. All other authors declare that they have no competing interests.

## Authors' contributions

BL and JL performed the literature search. BL, AS, MM, TK, JH, JP, KS and SG contributed to the study design. BL, JL and MK assisted with data collection. BL, JH, TK, AS and MM informed the data analysis. All authors contributed to the interpretation of study results as well as manuscript preparation. In addition, the final version of the manuscript was read and approved by all authors.

## Pre-publication history

The pre-publication history for this paper can be accessed here:

http://www.biomedcentral.com/1472-6963/12/30/prepub
